# Primary arthrodesis for diabetic ankle fractures using a modified retrograde femoral intramedullary locking nail combined with lateral plating, surgical technique, and early results of a pilot study

**DOI:** 10.1007/s00590-024-03947-1

**Published:** 2024-05-01

**Authors:** Amr A. Fadle, Wael El-Adly, Momen Ayman Fekry, Ahmed E. Osman, Ahmed A. Khalifa

**Affiliations:** 1 Department of Orthopedic, Assiut University Hospital, Assiut, Egypt; 2 Department of Orthopedic, Qena Faculty of Medicine and University Hospital, South Valley University, Kilo 6 Qena-Safaga Highway, Qena, 83523 Egypt

**Keywords:** Ankle arthrodesis, Diabetic, Ankle fracture, Intramedullary nail

## Abstract

**Purpose:**

We aimed to report the early results of performing acute ankle arthrodesis using a modified retrograde femoral intramedullary locking IMN concomitant with plating at the same setting for managing diabetic patients' acute ankle fractures.

**Methods:**

We prospectively included patients who presented acutely with ankle fractures, where hemoglobin A1C (HbA1C) on admission was > 7%, and the Adelaide Fracture in the Diabetic Ankle (AFDA) algorithm score was 5 or above. All patients were treated by acute ankle arthrodesis using a modified retrograde femoral IMN combined with lateral plating. Functional assessment was reported according to a modified American Orthopaedic Foot and Ankle Society ankle hindfoot scale (AOFAS), and complications were documented.

**Results:**

Six patients had an average age of 55.7 years (37–65). The average HbA1C on admission was 7.9 (7.3–9), and the average AFDA score was 7.3 (6–8). The average operative time was 79.2 min (70–90). All patients, except for one, achieved union at the arthrodesis site after an average of 10.3 weeks (8–14). After an average last follow-up of 9 months (6–12), the average modified AOFAS was 73.2 (82 to 62); four patients had an excellent score and one good. Complications developed in two, one deep infection after 2 weeks treated by metal removal and Ilizarov, and the other patient developed a stress fracture at the tibial end of the nail, which was treated by open reduction and internal fixation using a plate and screws**.**

**Conclusion:**

Using a modified femoral IMN combined with lateral plating is a promising technique to achieve ankle arthrodesis in diabetic patients with acute ankle fractures with acceptable outcomes; however, further studies with larger numbers are needed.

**Level of evidence:** IV

## Introduction

Ankle fractures in diabetic patients carry more complications risk compared to matched none diabetic patients, with an overall complication incidence of up to 47% compared to 15%, respectively [1–3]; furthermore, diabetes mellitus (DM) showed the highest odds of subsequent amputation compared to other risk factors [4].

Owing to the increased local and general risks associated with ankle fractures in diabetic patients, some surgeons suggested that conservative lines of management are safer; however, this turned out to be wrong over time, as more studies showed increased complications with conservative management and a higher risk for developing Charcot arthropathy [5–8].

Surgical fixation by open reduction and internal fixation (ORIF) was suggested to offer more rigid fixation with subsequent better outcomes and function; however, less than optimum fixation led to catastrophic failures, with a further need for revision surgery or ankle arthrodesis [3, 9, 10]. This led some surgeons to perform ankle arthrodesis acutely for managing ankle fractures in poorly controlled diabetic patients depending on various preoperative patients and fracture characteristics [11, 12], which was achieved using various fixation techniques, including retrograde tibial-talo-calcaneal (TTC) nail fixation, Ilizarov external fixator, ORIF using plates and screws, or combination of these techniques [3, 7, 12–14].

Manway et al. [7, 15] suggested that the concept of “super-construct” applied initially for Charcot arthropathy management to be applied for diabetics’ ankle fracture fixation, aiming at increasing the fixation strength and mechanical properties, planning surgical incisions with limiting deep dissection and extending the fixation beyond the injury zone. Furthermore, applying plates and screws concomitant with intramedullary nail (IMN) for increasing the rigidity of the fixation construct had been described thoroughly in the trauma literature, with good outcomes [16–18]. The current series aims to report the surgical technique and early results of performing acute ankle arthrodesis using a modified retrograde femoral IMN concomitant with lateral plating (as a super-construct) for managing acute ankle fractures in complicated diabetic patients.

## Methods

**Fig. 1:**
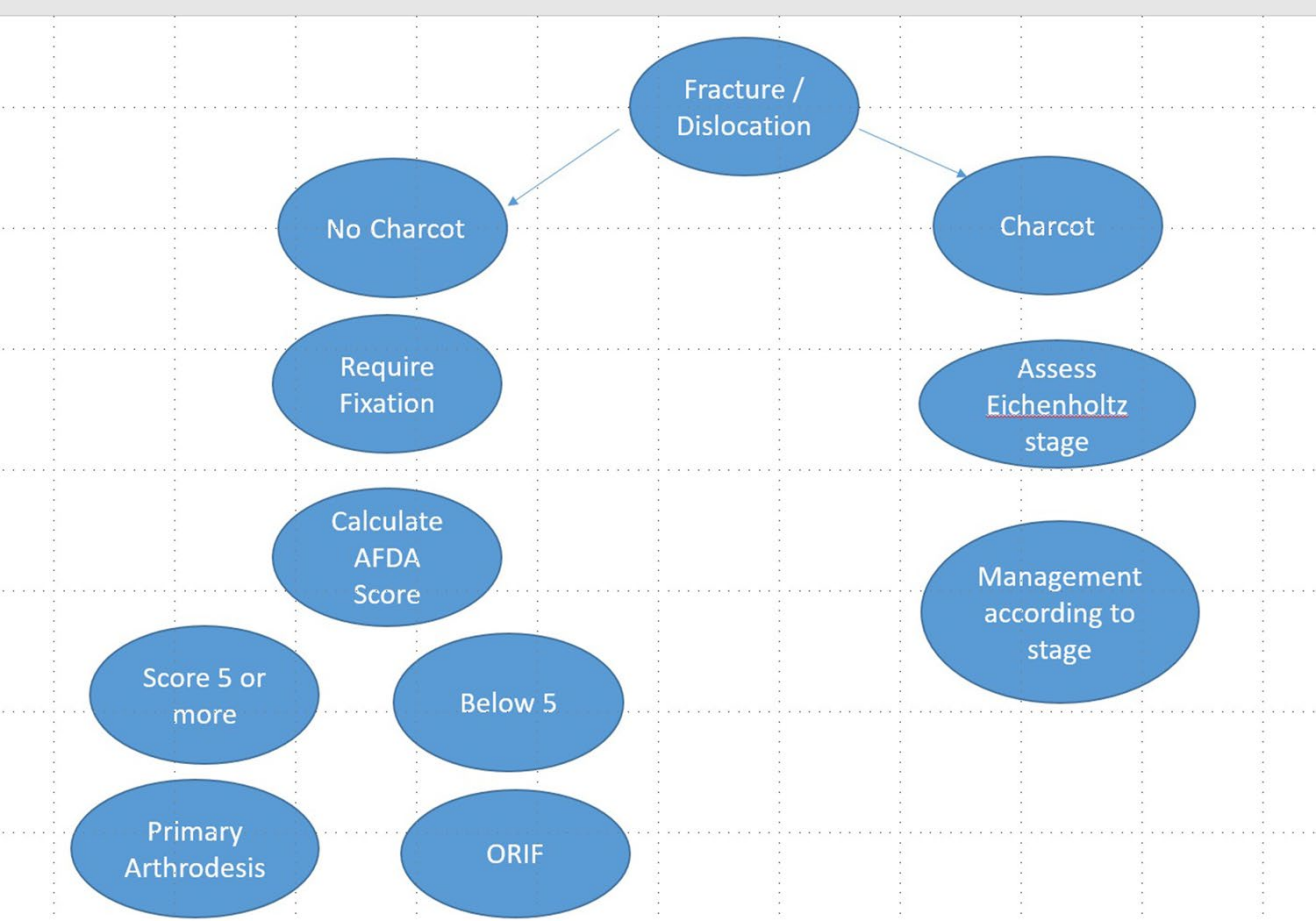
AFDA diagnostic and management Algorithm

After obtaining approval from our institution’s ethical committee (IRB No.: 17200615), diabetic patients who presented acutely (within a few days of the trauma) with ankle fractures between March 2021 and May 2022 were prospectively included. Patients who presented late had an active infection or had advanced Charcot arthropathy were excluded. This resulted in eight patients being included. All patients were evaluated clinically and radiologically. The blood glucose levels and hemoglobin A1C (HbA1C) values were evaluated on admission. The decision to perform acute ankle arthrodesis was based on the Adelaide Fracture in the Diabetic Ankle (AFDA) algorithm and score (Fig. 1, Table 1); (patients had fracture/dislocation not in Charcot state) then patients scored 5 or above [19], and if HbA1C on admission was > 7% [20], these were candidates for acute ankle arthrodesis.

**Table 1:** AFDA score

Two points	One point
Peripheral neuropathy/loss of peripheral sensation	1-Diabetic history of greater than 20 years
Presence of vasculopathy	2-Presence of diabetic nephropathy or retinopathy
Insulin dependence with poor compliance	3-Obesity
Previous or coincidence history of Charcot of any joint	4-Poor patient compliance

### Surgical technique

All patients were operated upon in a supine position under spinal anesthesia and tourniquet control, with the foot hanging out of the table. All ankles were approached through a direct lateral trans-fibular approach. The distal end of the fibula (distal to fracture site) was excised and kept to be used as a local graft, followed by preparing the ankle articular surface by removing the synovium and the articular cartilage from the distal tibia and the talus’s dome. Then, an initial stabilization of the ankle joint in the optimum arthrodesis position (we aimed for neutral dorsiflexion, 5–10° of external rotation, 5° of hindfoot valgus, and 5 mm of posterior talar translation) was achieved using multiple k-wires (which should be away from the expected nail guide wire track). We started with IMN fixation using a modified retrograde femoral IMN (Orthomed-Co., Egypt); the entry point was located 2 cm medial and posterior to the Calcaneocuboid joint, followed by inserting a guide wire under C-arm control. Gradual incremental intramedullary reaming was started until a chatter was felt, and then, an IMN smaller by 1 mm than the last reamer was used; in all cases, we used an IMN of 28 cm in length. After confirming the appropriate ankle position under C-arm control, the IMN was inserted and rotated in a manner enabling insertion of the calcaneal screws in a posterior-to-anterior direction so that we could use the longest screw possible. The calcaneal locking screws were inserted using the aiming device.

**Fig. 2:**
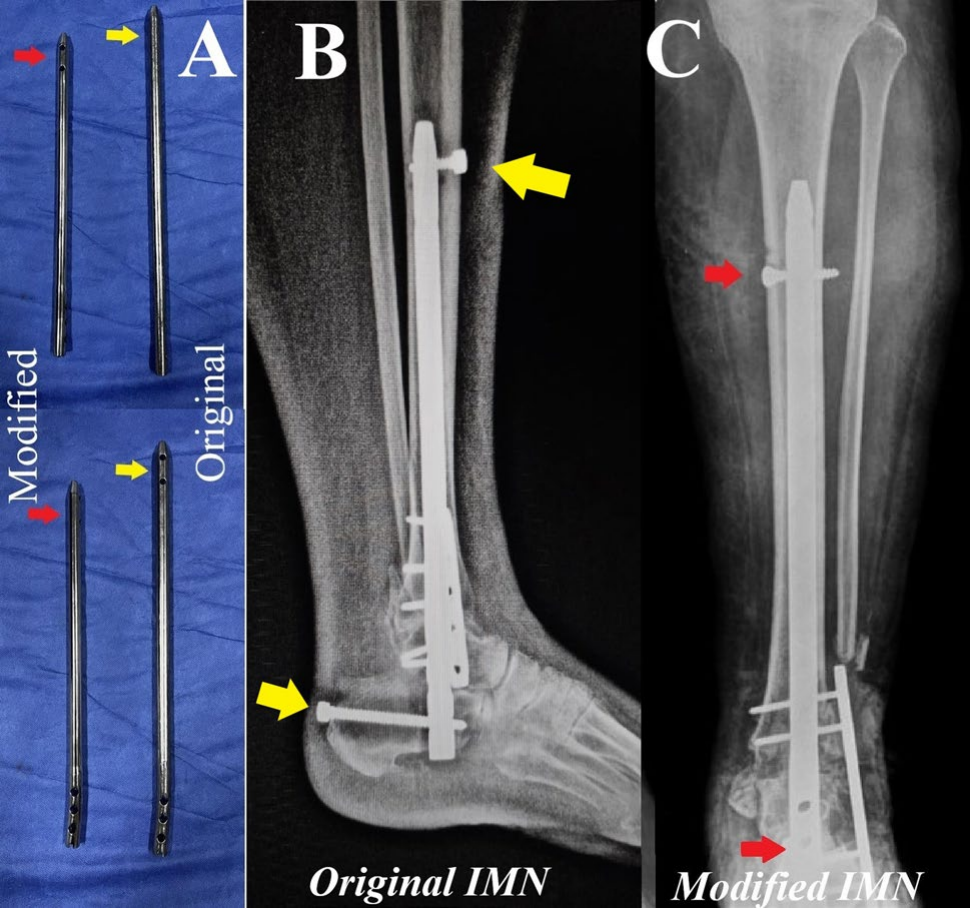
The modification introduced to the retrograde femoral IMN. **A** Comparison between the original (right) and the modified (left) IMN, where the proximal and distal locking screw holes became at 90 degrees to each other (red arrows) instead of being colinear (yellow arrows). **B** Radiographic image (lateral) showing the original IMN in place with the proximal and distal screws are colinear, showing the anterior prominence of the tibial locking screw (yellow arrows). **C** Radiographic image (anteroposterior) showing the modified IMN with the tibial locking screws fixed from medial to lateral (red arrows)

*The retrograde femoral IMN modification (*Fig. 2*):* The original nail design is formed of a proximal slightly curved end, which contains static and dynamic slots for the locking screws, and the distal end is straight and has two screw holes for distal locking screws insertion. In the original IMN, the screw holes (proximal and distal) are colinear, so when using this design aiming at achieving calcaneal end locking screws aiming posterior to anterior, this will obligate the surgeon to place the tibial end locking screws in an anterior to posterior position, which will be prominent anteriorly and could cause discomfort owing to the scares soft tissue cover at this area. We modified the orientation of the locking screw holes in the tibial end to 90° so that the tibial locking screws would be taken from medial to lateral and avoid being prominent anteriorly.

Before performing the tibial side locking of the nail, a small locked dynamic compression plate (DCP) was placed on the lateral aspect of the ankle with distal locking screws fixed to the calcaneus (posterior to the calcaneocuboid joint), and the dynamic compression function of the plate was used to help to achieve compression across the ankle arthrodesis site by inserting compression screws in the tibial portion of the plate. After securing the plate and screws, free-hand insertion of the tibial end locking screws of the IMN was performed.

**Fig. 3:**
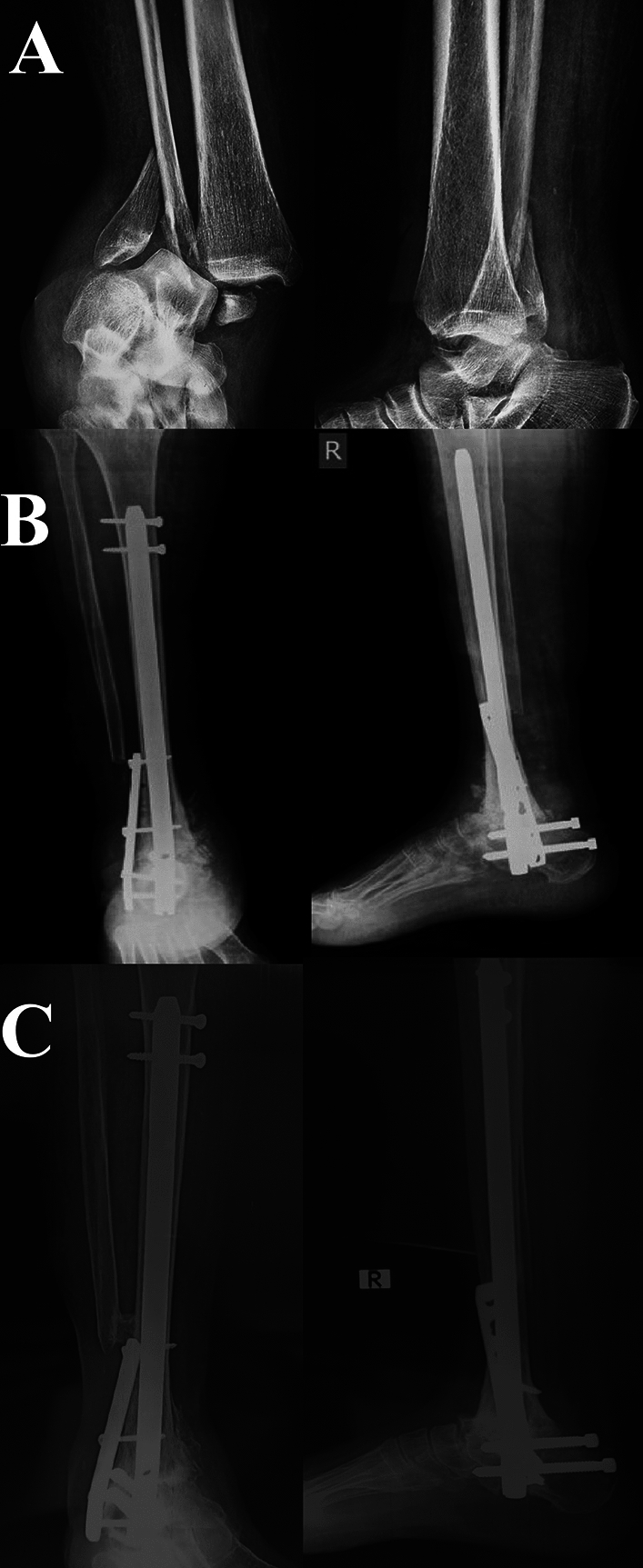
A female patient 63 years old, presented with ankle fracture dislocation (OTA/AO 44B3) (HbA1C: 7.7 and AFDA score 8). **A** preoperative radiographs. **B** Immediate postoperative radiographs. **C** At 12 months, follow-up radiographs showed stable implants and ankle arthrodesis

**Fig. 4:**
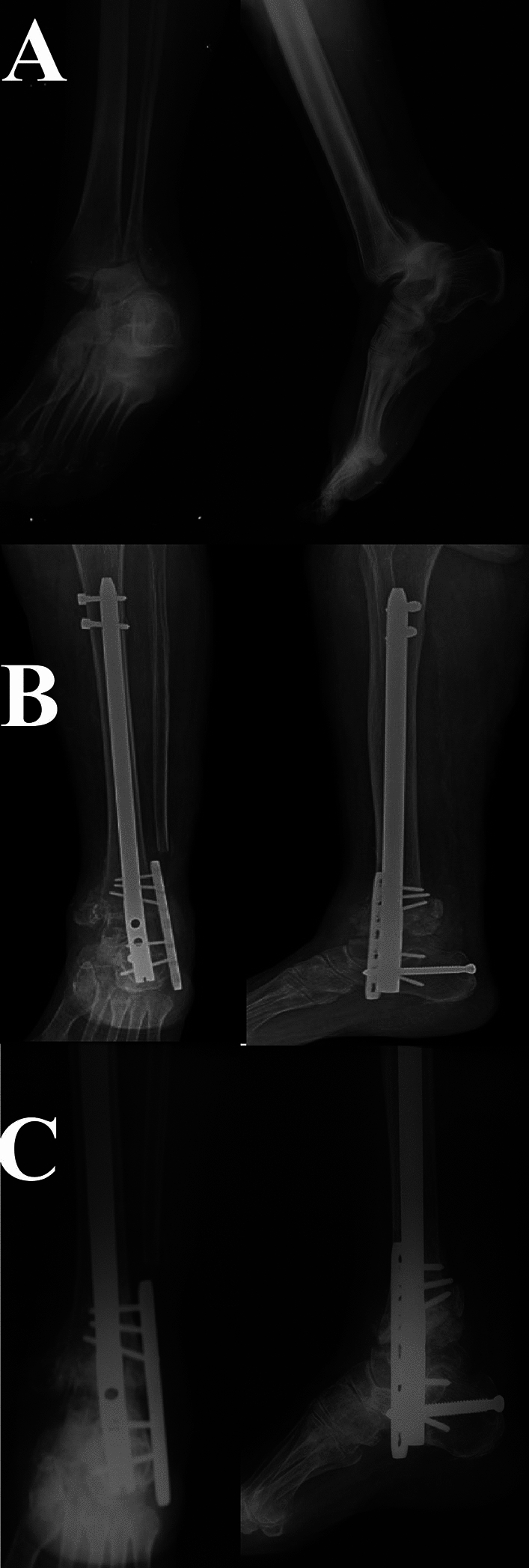
A female patient 55 years old presented with ankle fracture dislocation (OTA/AO 44B3) (HbA1C: 7.5 and AFDA score 7). **A** Preoperative radiographs. **B** Immediate postoperative radiographs. **C** At 10 months, follow-up radiographs showed stable implants and ankle arthrodesis

*Postoperative protocol:* Postoperatively, patients were placed in a posterior below-knee slab for eight weeks. After an average of 4 days, patients were discharged from the hospital, ensuring blood glucose level adjustment, monitoring for DVT development, and early postoperative infection. The follow-up visits were scheduled at 2 weeks (for sutures removal, wound assessment, and change of the posterior slab). Then, the patients were instructed to follow up every month (for wound assessment, complications development, and radiographic evaluation of union) till radiographic evidence of fusion, then every 2 months till the end of the first postoperative year (Figs. 3, 4). Functional assessment was performed according to a modified American Orthopaedic Foot and Ankle Society ankle hindfoot scale (AOFAS), with a total of 86 points instead of 100 points of the original scale (as 14 points representing the ankle and subtalar movement were deducted), we considered a score of 86 to 74 as excellent, 73 to 64 as good, 63 to 54 as fair, and less than 54 as poor [21, 22].

## Results (Table 2)

**Table 2:** Patients details

Patient no	Age (years)	Sex	HbA1C (on admission)	Fracture classification (OTA/AO)	AFDA score	operative time (minutes)	complication	Arthrodesis union (months)	Modified AOFAS
1	55	F	7.5	44B	7	75	No	8	82
2	62	M	7.4	44B	8	70	No	10	77
3	52	F	9	44C	7	80	Stress # tibia proximal to nail	12	78
4	37	F	8.5	44B	6	90	Infection after 2 weeks removal of nail and Ilizarov	10	64
5	65	F	7.3	44C	8	85	No	8	62
6	63	F	7.7	44B	8	75	No	14	76

HbA1C: Hemoglobin A1C, AFDA: Adelaide Fracture in the Diabetic Ankle, AOFAS: American Orthopaedic Foot and Ankle Society

Six patients having an average age of 55.7 years (37–65), five females and one male were available after an average last follow-up of 9 months (6–12), (one patient died of irrelevant causes, and one was lost during follow-up). Patients presented after trauma by an average of 2.8 days (1–5). According to the OTA/AO classification system [23], fracture classification was 44B and 44C in four and two patients, respectively. The average HbA1C on admission was 7.9 (7.3–9), while the average AFDA score was 7.3 (6–8). The same surgeon (senior foot and ankle surgeon) operated on all patients. The average operative time was 79.2 min (70–90). All patients, except for one, achieved union at the arthrodesis site after an average of 10.3 weeks (8–14). Functional assessment at the last follow up, according to the modified AOFAS, showed an average score of 73.2 (82–62), where four patients had an excellent score and one good. Complications developed in two patients, one with a bad blood glucose level control developed a deep infection after 2 weeks, necessitating removing all hardware and applying Ilizarov external fixator. The other patient developed a stress fracture at the tibial end of the nail (three months postoperatively after she started walking unsupported), which was treated by open reduction and internal fixation using a plate and screws, and eventually united without further complications.

## Discussion

Managing ankle fractures in diabetic patients requires a multi-disciplinary team approach, as besides the fracture, those patients could suffer from general as well as local complications owing to hyperglycemia, mainly peripheral vascular insufficiency and peripheral neuropathy, which could affect the outcomes and increase the risk of complications [3, 6, 24]. An increase in complications incidence of about 21-fold odds ratio after non-surgical management of ankle fractures in diabetic patients, as shown by Lovy et al. Furthermore, the authors reported 100% complication incidence in secondary ORIF after failed non-surgical management compared to 12.5% in patients treated primarily by ORIF [5].

Ankle arthrodesis is well described as a salvage procedure for managing Charcot arthropathy and severe pilon fractures [11, 25–27]. However, the literature describing its utilization in managing acute ankle fractures without a concomitant Charcot arthropathy is scarce [10, 12, 28].

In the current series, to decide which patient is a candidate for primary ankle arthrodesis after an acute ankle fracture, we decided based on the HbA1C level on admission, as Liu et al. showed that diabetic patients with ankle fracture treated by ORIF who had an HbA1C above 6.5 are more prone to worse functional and radiological outcomes, with a higher complication rate [20]. Furthermore, we followed a score over 5 according to the Adelaide Fracture in the Diabetic Ankle (AFDA) algorithm and score as proposed by Yee et al. [19]. However, this algorithm was criticized for not including the fracture characteristics and/or classification (open vs. closed, dislocated vs. not), which could affect the soft tissue status and, subsequently, the management decision [12]. In our study, we noticed that peripheral neuropathy is the main pathology that exists in all of our six patients and we can consider it the most important complication of diabetes which may affect the decision or the results of the surgery.

In a retrospective study by Grote et al., 13 diabetic patients presented with acute ankle fractures managed by acute TTC arthrodesis using hindfoot nails, although a high incidence of complications reaching up to 75% was reported; however, after an average follow-up of 297 days, 89.9% of the included patients had fracture union and stable lower extremity. The authors reported that the decision to perform arthrodesis for diabetic patients with ankle fractures was based on individual surgeons’ judgment considering the patient’s general condition and the severity of the injury; however, there was no formal protocol or guideline to include or exclude patients. However, the authors reported considering diabetic complications (such as organ damage) and calculating AFDA scores (cut of the value of 5) (Table 3) [12].

**Table 3:** Patients AFDA Score in detail

	Peripheral Neuropathy	Vasculopathy	Insulin dependence (poor compliance)	Previous Charcot arthropathy	History of 20 years of DM	History diabetic nephropathy, retinopathy	Obesity	Poor patient compliance
Patient 1	✓		✓	✓			✓	
Patient 2	✓	✓	✓		✓		✓	
Patient 3	✓		✓		✓	✓	✓	
Patient 4	✓	✓			✓			✓
Patient 5	✓	✓	✓		✓		✓	
Patient 6	✓		✓	✓			✓	✓

Bold pathology (2 points), other pathology (1 point)

In the current series, we performed open surgical preparation of the articular surface aiming at achieving sold arthrodesis; however, TTC nailing without joint preparation was described by Ebaugh et al. while managing 27 patients having a mean age of 66 years with ankle fractures and concomitant complicated diabetes (defined as having neuropathy, nephropathy, and/or peripheral vascular disease), the mean operative time was 73 min, the authors reported limb salvage rate of 96%, and fracture union rate of 88%, although they reported complications incidence of 18.5% (mainly infection); however, it did not include non-union, malunion, or development of Charcot arthropathy, the decision not to prepare the joint surfaces was to safe operative time and surgical incision in their high risk and low demand patients [29].

The use of a retrograde femoral IMN for ankle arthrodesis was described by Pinzur et al. [11], where the authors used it in the ankle arthrodesis of nine patients presented with Charcot arthropathy; all patients achieved fusion after an average of 10.5 weeks, the authors reported no complications related to the IMN, especially stress fractures. Furthermore, Powers et al. reported their results of performing 109 ankle arthrodesis for various indications using femoral IMN; they reported a union rate of 81.7%, and the authors stated that outcomes were comparable to other methods [30].

The technique we are proposing carries various advantages. First, as it is an open technique (similar to most of the published studies), it enables the preparation of the articular surface for better bone coaptation and a higher possibility of fusion. Second, we adopted the concept of “super-construct” for rigid fixation by combining the plate on the lateral side with the IMN, aiming at improving the stability of the construct, especially the rotational stability, as the nail alone is better in axial stability but weak in rotational stability [18]. Third, using a locked small DCP offered some advantages; the dynamic effect of the plate was used to achieve compression at the arthrodesis site before IMN distal locking, being a low profile enabled easy closure of the surgical wound, and the locking screws had better purchase in weak osteoporotic bone. Fourth, the modification we introduced to the IMN avoids the anterior prominence of the screws on the tibial side. Furthermore, it enabled us to insert the calcaneal screws in a posterior-to-anterior direction and use the longest possible screw, leading to better purchase and stability. Last, regarding the cost issues, although we did not perform a detailed cost analysis, considering only the cost of the implants, in our country, using a specialized TTC nail costs about fivefold the cost of a retrograde femoral IMN.

The limitations of the current study are the small number of included patients, and there was no comparative group; however, this could be attributed to the high selectivity of the patient to be included in this technique. Second is the relatively short follow-up period which was comparable to previous studies and suitable when considering a complete union of arthrodesis site as an endpoint. Last is the lack of a biomechanical study to confirm the superiority of our proposed construct over using IMN alone.

## Conclusion

Deciding on performing acute ankle arthrodesis in diabetic patients with acute ankle fractures should be considered in certain situations, and robust fixation is paramount for achieving ankle arthrodesis. The technique we introduced using a modified retrograde femoral IMN combined with lateral plating is promising and provides an effective option for obtaining ankle arthrodesis; however, the lower cost as an advantage should be deeply investigated, and further comparative studies including a larger patient number are needed to confirm the proposed advantages of this technique.

## Data Availability

All the data related to the study are mentioned within the manuscript; however, the raw data are available with the corresponding author and will be provided up on a written request.

## Abbreviations

DM: Diabetes mellitus
ORIF: Open reduction and internal fixation
TTC: Retrograde tibio-talo-calcaneal
IMN: Intramedullary nail
HbA1C: Hemoglobin A1C
AFDA: Adelaide Fracture in the Diabetic Ankle
DCP: Dynamic compression plate
AOFAS: American Orthopaedic Foot and Ankle Society

## Declarations

### Study setting

Foot and Ankle surgery unit, Orthopaedic department, Assiut University Hospital, Assiut, Egypt.
